# Mast Cells in Viral, Bacterial, and Fungal Infection Immunity

**DOI:** 10.3390/ijms20122851

**Published:** 2019-06-12

**Authors:** Adrian M. Piliponsky, Manasa Acharya, Nicholas J. Shubin

**Affiliations:** 1 Departments of Pediatrics and Pathology, University of Washington, Seattle, WA 98195, USA; 2 Center for Immunity and Immunotherapies, Seattle Children’s Research Institute, Seattle, WA 98101, USA; manasa.acharya@seattlechildrens.org (M.A.); nicholas.shubin@seattlechildrens.org (N.J.S.)

**Keywords:** mast cells, infection, flavivirus, cecal ligation and puncture, *Candida albicans*, innate immunity, inflammation

## Abstract

Mast cells are granule-rich immune cells that are distributed throughout the body in areas where microorganisms typically reside, such as mucosal tissues and the skin, as well as connective tissues. It is well known that mast cells have significant roles in IgE-mediated conditions, such as anaphylaxis, but, because of their location, it is also thought that mast cells act as innate immune cells against pathogens and initiate defensive immune responses. In this review, we discuss recent studies focused on mast cell interactions with flaviviruses and *Candida albicans*, and mast cell function in the cecal ligation and puncture model of sepsis. We selected these studies because they are clear examples of how mast cells can either promote host resistance to infection, as previously proposed, or contribute to a dysregulated host response that can increase host morbidity and mortality. Importantly, we can distill from these studies that the contribution of mast cells to infection outcomes depends in part on the infection model, including the genetic approach used to assess the influence of mast cells on host immunity, the species in which mast cells are studied, and the differential contribution of mast cell subtypes to immunity. Accordingly, we think that this review highlights the complexity of mast cell biology in the context of innate immune responses.

## 1. Introduction

Mast cells are predominantly found at host-environment junctures, such as the skin and intestinal mucosa. Due in part to their location, it has been hypothesized that mast cells can act as innate immune cells that detect pathogens and initiate immune responses that promote pathogen clearance. In support of this hypothesis, there is an extensive amount of work using murine and human mast cells isolated *ex vivo*, or differentiated from stem cells, as well as mast cell lines, showing that mast cells can recognize pathogens via expression of pathogen recognition receptors, including Toll-like receptors (TLR) and nucleotide-binding oligomerization domain (NOD) proteins, and by binding to antibodies with Fc receptors [1,2,3]. A recent study also suggested that mast cell activation via the Mas-related G protein-coupled receptor (GPCR) member X2 (MRGPRX2) may enhance host innate immunity against bacteria and accelerate infection resolution [4]. It should be noted that *in vitro* studies have shown that expression and function of these receptors may vary among species and mast cell phenotypes [5,6]. For example, MrgprB2, the mouse orthologue of MRGPRX2 [7], is found exclusively in connective tissue mast cells, making this receptor potentially relevant to skin infections, where mast cells are very abundant.

A protective role for mast cells in the host immune response against pathogens was initially reported for infections with parasites. Specifically, Crowle PK used Kit*^W/W-v^* *c-kit* mutant mast cell deficient-mice to demonstrate that engraftment of these mice with mucosal mast cells contributes to the rejection of *Nippostrongylus brasiliensis* [8]. Similar approaches were subsequently used to show that mast cells can also be protective during bacterial infections that affect different compartments in the host. For example, Carlos D. et al. showed that the adoptive transfer of wild-type mast cells into TLR2-deficient mice restores the ability of these mice to control airway infection with *Mycobacterium tuberculosis* (*Mtb*) and promote the recruitment of myeloid cells associated with granuloma formation [9]. These observations suggest that mast cells have an important role during the early, as well as the late, phase of mycobacterium infection. Despite this and other studies (summarized in a recent review by Garcia-Rodriguez K.M. et al. [10]) supporting a protective role for mast cells, the contribution of these cells to immunity against *Mtb* infection remains largely unknown. 

In addition to *Mtb*, we recently showed that mast cells also play an important role in Group B *Streptococcus* (GBS) infections. GBS are Gram-positive bacteria that frequently colonize the lower genital tract of healthy women and cause infections of the amniotic cavity and uterus, which can result in preterm birth, septic abortion, and postpartum endometritis [11]. By using *c-kit*-independent mast cell-deficient mice, we were able to show that mast cell deficiency is associated with enhanced bacterial burden, decreased neutrophil mobilization, and decreased immune responses during systemic GBS infection. Moreover, hyperpigmented GBS strains showed increased persistence in mast cell-deficient mice when compared with mast cell-proficient mice in a vaginal colonization model [12]. In a more recent study, we were able to provide evidence that mast cell chymase protects against GBS dissemination and pre-term labor via proteolysis of the host extracellular matrix protein, fibronectin [12]. 

It has also been shown that mast cells can be protective against skin infections. As mentioned earlier in this review, Arifuzzaman M. et al. recently showed that skin mast cell activation by mastoparan, an IgE-independent mast cell secretagogue, can enhance bacteria clearance in a mouse dermonecrotic *Staphylococcus aureus* infection model via neutrophil recruitment [4]. Moreover, mast cell activation also induces the migration of antigen-presenting dendritic cells to draining lymph nodes, enhancing protection against a secondary infection [4]. Reports on the protective role of mast cells during viral infections are much more limited in number and scope, except for studies focused on infections that mainly affect the skin, such as those caused by vaccinia virus (VV) and flavivirus (see expanded section below). VV is a member of the *Orthopoxvirus* genus of the Poxviridae family, which includes variola (smallpox) virus, monkeypox virus, cowpox virus, and ectromelia virus. The protective role of mast cells in skin infections with VV was investigated in depth by Di Nardo’s group, who showed that mast cells can sense VV via the sphingosine-1-phosphate (S1P) G-coupled receptor (S1PR2), leading to degranulation and killing of VV via the release of antimicrobial peptides, such as cathelicidin [13].

The remarkable expansion of mast cell research into different models of infection, including bacteria, fungi, viruses, and parasites, revealed that mast cell responses can also be pro-pathogenic [14]. Abnormalities associated with the *c-kit* mutation and/or mouse backgrounds may have influenced the infection outcomes in some of these studies, but, in others, more than one strain of mast cell-deficient mice, including *c-kit*-dependent and *c-kit*-independent mast cell-deficient, were used, strengthening the evidence that mast cells can contribute to dysregulated responses against pathogens that lead to increased morbidity and mortality. This leads us to the question: how does the pathogenicity of an infectious agent and/or the host’s response against it influence the contribution of mast cells to infection?

As an attempt to answer this question, this review focuses on recent advances in the field of mast cells and innate immunity that highlight the beneficial and detrimental roles of mast cells in different models of infection caused by viruses, bacteria, and fungi. For this purpose, we selected specific pathogens or models of infection where the beneficial and detrimental roles of mast cells were investigated. Specifically, we will discuss mast cell responses to flavivirus infections, mast cell functions in the cecal ligation and puncture (CLP) model of sepsis, and mast cell interactions with *Candida albicans*, which are examples of viral, bacterial, and fungal infections, respectively.

## 2. Mast Cells and Flavivirus Infections 

Many viruses that are of interest to humans belong to the *Flavivirus* genus in the Flaviviridae family. These include arthropod-borne viruses (arboviruses) that are delivered by mosquitoes and ticks, such as dengue virus (DENV), yellow fever virus (YFV), West Nile virus (WNV), Japanese encephalitis virus (JEV), Zika virus (ZIKV), and Tick-borne encephalitis virus (TBEV). These viruses represent a threat to populations, particularly in areas where people are naïve to the infection. Additionally, due to climate change and the increased movement of people, more and more sudden outbreaks of these viral infections have been observed outside endemic areas. Given their increasing significance to human health and the lack of treatment options, these viruses represent a challenge for the development of novel direct antiviral medications [15].

There is an extensive amount of literature focused on the beneficial and detrimental roles that mast cells can play during DENV infections, which is one of the most prevalent human viral maladies of the twenty-first century, as nearly 390 million infections have occurred annually worldwide in recent years [16]. 

### 2.1. Beneficial Effects of Mast Cells in DENV Infections 

DENV is an arthropod-borne virus (arbovirus). DENV is usually transmitted by the bite of infected mosquitoes that inject DENV particles into the skin, which then proceed to infect the surrounding cells, such as dendritic cells [17]. Infected dendritic cells can become activated and migrate to draining lymph nodes where the infection can then be amplified [18]. Type I interferon (IFN). such as IFNα, is a critical mediator of the anti-DENV response, inhibiting not only viral replication, but also contributing to the activation of cells with the ability to kill DENV-infected cells, such as natural killer (NK) cells and T cells [19,20,21]. However, the cell types involved in initial surveillance for DENV are less well defined. Mast cells are prevalent in the skin and, therefore, are likely to be among the first immune cells to encounter DENV. 

**Figure 1:**
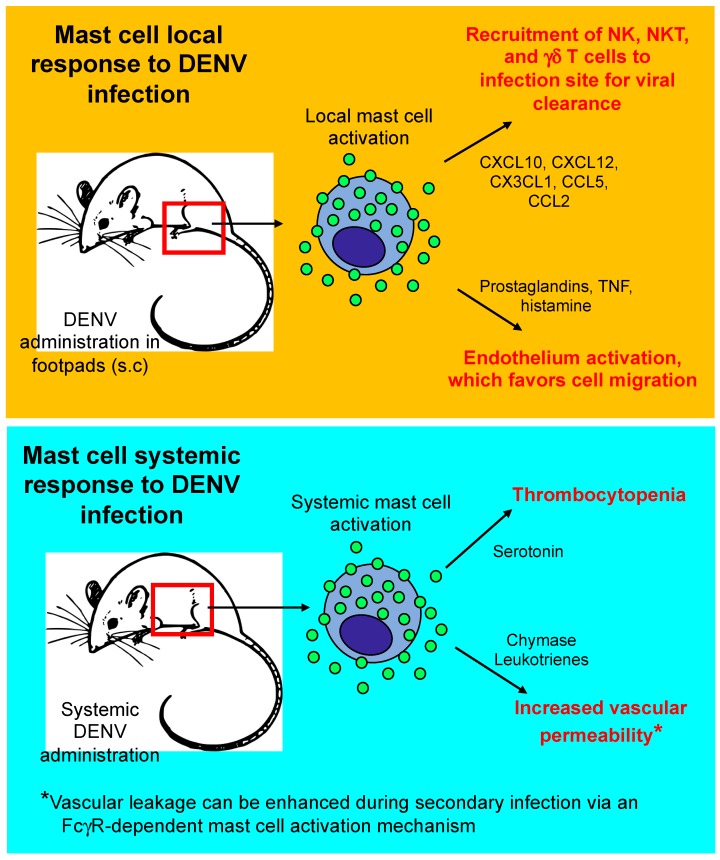
Mast cell response to local and systemic dengue virus infection. DENV, dengue virus; TNF, tumor necrosis factor; FcγR, Fc gamma receptor; NK, natural killer cells; NKT, natural killer T cells; γδ T cells, gamma delta T cells; s.c., subcutaneous; CXCL10, (C-X-C motif) chemokine ligand 10; CXCL12, (C-X-C motif) chemokine ligand 12; CX3CL1, (C-X3-C motif) chemokine ligand 1; CCL5, chemokine (C-C motif) ligand 5; CCL2, chemokine (C-C motif) ligand 2.

How might mast cells detect DENV? In vitro studies with mast cell-like rat basophilic leukemia-2H3 (RBL) cells have shown that cellular sensors for viral RNA, such as toll-like receptor (TLR)3, melanoma differentiation-associated protein (MDA)5, and retinoic acid-inducible gene (RIG)-I, help to trigger an anti-DENV transcriptional program in these cells [22]. This anti-DENV transcriptional program includes an up-regulation in the type I interferon response and the production of chemokines, such as CXCL12 and CX3CL1, with the ability to promote the recruitment of cytotoxic cells, such as NK cells, which can potentially eliminate DENV-infected cells [23]. In vivo evidence for the latter was first shown by St. John A.L. et al. [22]. By using Kit*^W-sh/W-sh^* mast cell-deficient mice and Kit*^W-sh/W-sh^* mast cell-deficient mice reconstituted with wild-type mast cells, St. John A.L. et al. demonstrated that mast cells can inhibit viral spread from the inoculation site in the foot pad to the draining lymph node via the recruitment of NK cells and T cells bearing NK cell markers into the infected tissues [22]. A more recent study identified γδ T cells as the first T cell subset to respond to mast cell-driven inflammation and implicated these cells in DENV host defense [24] (Figure 1). 

### 2.2. Detrimental Effects of Mast Cells in DENV Infections

DENV infection can be asymptomatic or self-limited. About 25% of patients develop very minor symptoms, including a self-limited febrile illness accompanied by mild-to-moderate hematological and biochemical abnormalities. A small proportion of infected patients develop clinically relevant complications. These include systemic vascular leak syndrome, which can lead to dengue shock syndrome (DSS), and coagulation/bleeding abnormalities that are associated with dengue hemorrhagic fever (DHF), and hepatic or neurological organ dysfunction. These complications were reported to be associated with increased mast cell activation, as well as increased tryptase and chymase plasma concentrations [25,26,27], leading to the hypothesis that mast cells play a detrimental role in more severe forms of DENV infection. 

A detrimental contribution of mast cells in the development of DENV complications was also demonstrated by using Kit*^W-sh/W-sh^* mast cell-deficient mice that were systemically infected with DENV. These mice exhibited a less pronounced increase in vascular permeability and platelet loss than wild-type mice [26]. Similar findings were reported with the use of ketotifen, a drug that can suppress mast cell function without producing a broad immunosuppressive effect in the host immune response against DENV [28]. A reduction in the severity of mouse vasculopathy was also observed with both pharmacological blockade of leukotrienes and inhibition of chymotryptic activity, providing evidence that mast cell-specific mediators, such as chymase and leukotrienes that are released by mast cells and/other cells, contribute to vascular permeability in a more severe form of DENV infection [26]. Interestingly, a more detailed mechanism for the ability of chymase to cause vascular permeability during flavivirus infection was recently proposed for JEV, a neurotropic flavivirus that can cause severe neurologic deficits. This study showed that chymase is released from mast cells upon exposure to JEV and can increase brain endothelial barrier permeability via cleavage of tight junction molecules, leading to increased central nervous system infection with JEV and worsened morbidity and mortality [29]. In addition to chymase and leukotrienes, it was recently demonstrated that mast cell-derived serotonin can cause thrombocytopenia in a severe model of DENV-induced disease [30].

Overall, these studies point to the relevance of mast cell degranulation and release of pre-form mediators in the development of vasculopathy and thrombocytopenia associated with severe DENV-induced disease. Notably, mast cell degranulation was also one of the first changes observed in mast cell function when exposed to a virus in vitro [31]. However, it is still unclear how viruses can directly induce mast cell degranulation. Our poor understanding of the mechanism by which viruses induce mast cell degranulation may be explained in part by the amount of disparate data generated on this specific mast cell function with cells obtained from different species, and from in vitro versus in vivo models of infection in which mast cells from the same species were examined. In the specific case of DENV, direct degranulation of primary mouse mast cells in response to DENV has not been reported, but extensive mast cell degranulation was observed at the inoculation site in mice and non-human primates [22] suggesting indirect mast cell activation via mediators generated during infection. In fact, connective tissue mast cells express functional receptors for complement components [32], and these mediators are found at high concentrations during DENV infections [33]. In contrast to mouse mast cells, human skin mast cells can degranulate when exposed to DENV. Moreover, it has been shown that DENV can accumulate in human skin mast cell granules and they may have the capacity to infect human dermal endothelial cells upon degranulation [34].

The severe form of DENV infection occurs most often in individuals experiencing secondary dengue virus infections [35] and, in this disease context, the extent of mast cell activation and degranulation seems be more consistent among mast cells from different species. A potential role for mast cells in the pathogenesis of dengue virus-induced disease caused by secondary infections was first reported by Sanchez L.F. et al., showing that DENV can induce degranulation of mast cells sensitized with mouse anti-DENV antiserum [36]. Further, King C.A. et al. showed that KU812 cells, a human mast cell/basophil cell line, are more permissive to infection and release higher concentrations of cytokines, such as IL-1β and IL-6, in response to antibody-enhanced dengue virus infection [37,38]. In subsequent studies, the same group was able to replicate these responses using HMC-1 cells, a human mast cell line, and cord blood-derived mast cells [38], and showed that human mast cells can also release chemokines that can recruit additional immune effector cells, such as CCL3, CCL4, and CCL5, and factors that can activate endothelial cells and potentially contribute to disease severity, such as tumor necrosis factor (TNF) [39]. Mechanistically, these responses seem to occur in an FcγRII-dependent manner [40,41], at least in vitro (Figure 1).

## 3. Mast Cells in the Cecal Ligation and Puncture (CLP) Model of Sepsis

**Figure 2:**
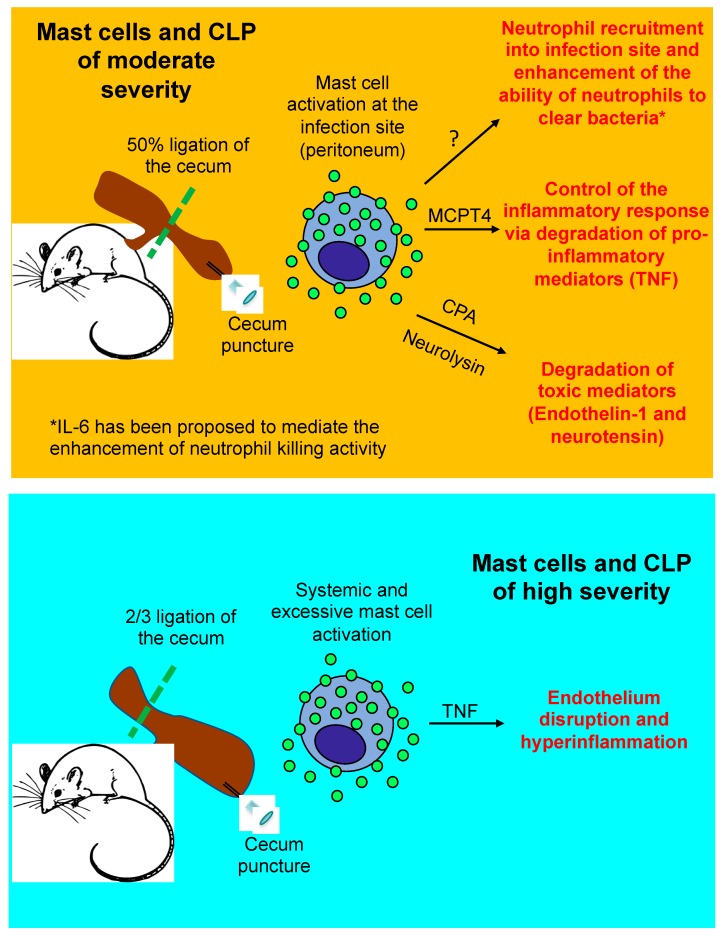
Mast cell response and its effects on the outcomes of cecal ligation and puncture of moderate and high severity. TNF, Tumor necrosis factor; MCPT4, mast cell protease-4; and CPA, carboxypeptidase A; CLP, cecal ligation and puncture.

CLP represents a model of traumatic or iatrogenic intestinal damage, which then results in a polymicrobial infection of the peritoneum. CLP is one of the most studied models where the contribution of mast cells to innate immunity against bacteria has been explored. This model involves ligation of the cecum, immediately below the ileocecal valve, to produce a distal ischemia, which is followed by a needle puncture of the ligated cecum. CLP severity can be modified depending on the location of the ligation, needle size, and/or puncture number. We and others have shown that 50% ligation of the cecum and one puncture with a 22G needle causes a local infection that resolves in 48 h and causes <50% mortality in wild-type mice (moderately severe CLP). Outcomes from genetic/or pharmacological manipulations in moderately severe CLP can reveal new pathways that influence bacteria clearance and/or inflammation in this model. Conversely, the response to 70% cecum ligation and one puncture with a 22G needle (severe CLP) closely mimics human severe sepsis progression, including bacteremia, systemic inflammatory response syndrome, multi-organ dysfunction, and >80% mortality in wild-type mice [42,43]. Outcomes from genetic or pharmacological manipulations in the severe CLP model usually affect the magnitude and/or duration of the hyper-inflammatory response to infection, rather than bacteria clearance. The contribution of mast cells to the outcomes of these two models will be discussed next and are summarized in Figure 2.

### 3.1. Beneficial Role of Mast Cells in Moderately Severe CLP

Mast cells play a vital role in the protection against enterobacteria infection in moderately severe CLP, as shown in a landmark study published in 1996 [44]. 

Bacteria detection via TLRs in mast cells, and more specifically via TLR4, is required for mast cell protection in this CLP model [45]. However, although TLRs activate mast cells, *in vitro* studies involving TLR ligands showed that mast cells do not degranulate in response to TLR stimulation. As such, it can be argued that degranulation is not required for the protective effect of mast cells during bacterial infections. In fact, we recently demonstrated that mast cell protease (MCPT)4, the functional mouse homologue of chymase [46], protects against systemic infection caused by a strain of Group B *Streptococcus* that does not induce beta hexosaminidase release, a marker of mast cell degranulation. However, mediators typically observed in pre-formed mast cell granules, including histamine and mast cell proteases, were observed in mice following CLP [47,48,49,50]. Moreover, peritoneal mast cells showed morphological evidence of degranulation during LPS-induced endotoxemia [50]. One explanation for these observations is that CLP and LPS administration leads to increased levels of endogenous peptides (e.g., complement, endothelin-1, and neurotensin) that induce mast cells degranulation [48,51,52]. Moreover, mast cell activation can also be modulated by factors that are bacteria-unrelated. For example, the protective effects mediated by mast cells in CLP can be enhanced by growth factors, such as stem cell factor (SCF) [53].

How do mast cells protect the host against bacteria? So far, it has been proposed that mast cells indirectly contribute to bacterial clearance by initiating the recruitment of inflammatory cells to the infection site [51,54,55]. Moreover, it has been shown that mast cell-derived mediators, such as MCPT6 [56] and IL-6 [57], are protective against *Klebsiella pneumoniae*, and that mast cell-derived TNF can amplify the inflammatory response against uropathogenic *Escherichia coli* [58]. However, how mast cells promote the recruitment of inflammatory cells into the infection site (peritoneum) after moderately severe CLP remains unclear. By using TNF-deficient mice [53] and TNF-neutralizing antibody-treated mice [59], it has been shown that this cytokine can have protective functions during CLP, and that such TNF-dependent effects may include the enhancement of neutrophil recruitment and/or function, as well as the promotion of bacterial clearance. Accordingly, it was hypothesized that mast cell-derived TNF also plays a protective role in the CLP model. However, by using knock-in mice, in which only mast cells do not produce TNF, we clearly demonstrated that mast cells are not the main cell source of TNF that is required to trigger inflammation in response to infection induced by moderately severe CLP [55]. Instead, we recently showed that basophil-derived TNF is required to enhance the ability of neutrophils and macrophages to clear bacteria in moderately severe CLP [60]. 

IL-6 also has been proposed as a mast cell-derived cytokine that can contribute to a positive outcome after CLP. In contrast to TNF, mast cells have been shown to contribute to increased IL-6 levels at the infection site (peritoneum) at very early stages following CLP. More importantly, it has been shown that mast cell-derived IL-6 significantly contributes to mouse survival after CLP [57]. Although it has been shown that the pro-inflammatory properties of IL-6 can enhance bacteria clearance during infection [61,62], mast cell-derived IL-6 does not protect mice from CLP by this mechanism [57]. The mechanisms by which mast cell-derived IL-6 protects in CLP remains largely unknown. 

Mast cell granules also contain many proteases that are thought to be specific to mast cells, including chymase, tryptase, and mast cell carboxypeptidase A3 (CPA3). Notably, the substrate specificity of these proteases have been conserved for over 150–200 million years [63,64], suggesting important roles for these proteases in innate immunity. There is evidence that mast cell-specific proteases can directly induce inflammatory cell mobilization to an infection site. For example, MCPT6, the mouse homologue of tryptase [65], is required for the recruitment of neutrophils to efficiently combat *K. pneumoniae* at the infection site [56]. Moreover, some studies seem to indicate that chymase can act as a potent chemoattractant for myeloid cells to induce neutrophil mobilization in vitro and in vivo [66,67]. Despite this evidence, it is unknown whether MCPT4 and MCPT6 can have direct effects on the recruitment of inflammatory cells into the peritoneum following moderate CLP; however, there is evidence that mast cell proteases can directly kill bacteria. More specifically, intracellular IL-15 expression in mast cells limits their MCPT2 mRNA levels, resulting in diminished chymotrypsin-like activity in mast cells, reduced mast cell-mediated defense against Gram-negative bacteria, and reduced survival of mice following CLP [68]. Except for this report, most of the studies on the contribution of mast cell proteases to CLP have focused on how these mediators can influence the host response to infection and prevent sepsis development. For instance, bacterial infections can lead to the production of endogenous mediators associated with sepsis pathophysiology, such as blood pressure dysregulation. For example, endothelin-1 [52] and neurotensin [48] are peptides that can cause vasoconstriction and hypotension in sepsis, respectively, and can be down-regulated by CPA and the non-specific mast cell protease, neurolysin, via proteolytic cleavage [48,69]. 

Unsurprisingly, it has been difficult to identify mast cell mediators that contribute to protection in moderate CLP by enhancing the inflammatory response. CLP is a complex polymicrobial infection model in which different mediators may have redundant roles. As such, knocking-out a specific mediator in mast cells may not lead to a different phenotype or outcome following CLP, as a different mediator with an overlapping function may compensate for its effect. Additionally, deletion of mast cells and their mediators in mice with *c-kit* mutations can be masked by the global effects from the c-kit mutation itself, such as changes in neutrophil numbers. To help remedy this issue, *c-kit*-independent mast cell-deficient mice have been developed and have already helped demonstrate how mast cells may contribute to bacterial-infection induced inflammation. For instance, diphtheria toxin A-treated, *Mcpt5*-Cre; iDTR^+^ mice lead to connective tissue mast cell ablation. Endotoxemia studies with these mice showed that mast cells and CXCL1/2 contribute to neutrophil recruitment into the peritoneal cavity following intraperitoneal LPS injection [50]; however, the beneficial role of mast cell-derived CXCL1/2 in CLP is still unclear. 

### 3.2. Detrimental Role of Mast Cells in Severe CLP 

The high-severity CLP model is the most commonly used murine sepsis model, as it resembles the cytokine-storm-mediated response to severe infection and induces multiple organ dysfunction syndrome and a high mortality rate (over 50%), which are associated with severe sepsis. Studies using the high-severity CLP model were among the first to show that mast cells can be detrimental following severe bacterial infection. 

In the high-severity CLP model, a main indicator of a dysregulated host response to infection is hyper-inflammation, which can lead to severe sepsis and shock, irrespective of the host’s ability to clear bacteria. Notably, we observed that mast cell-derived TNF significantly contributes to hyper-inflammation and death following severe CLP, using a mast cell knock-in model [55]. We confirmed these observations using a mouse in which TNF was only ablated in mast cells (unpublished data), generated by crossing a mouse that had Cre recombinase expressed under the control of the CPA3 promoter (Cpa3-Cre mouse) [70] with a TNF “floxed” mouse [71]. Furthermore, we observed that MCPT4-deficient mice [49] were impaired in their ability to down-regulate TNF levels following moderate CLP (as MCPT4 can proteolytically degrade TNF), which caused them to exhibit inflammation and mortality levels similar to those observed in wild-type mice following severe CLP [49]. A detrimental role for mast cells in severe CLP was also observed by Dahdah A. et al. In this study, the authors found that mast cell-derived IL-4 had immunosuppressive effects on the ability of macrophages to phagocytose bacteria, which they theorized is a potential mechanism by which mast cells promote mortality in severe CLP [72]. The reasons for the discrepancies observed between studies in how mast cells contribute to severe CLP outcomes remain largely unknown. 

Why mast cells exert a detrimental effect following severe but not moderate CLP is unclear. A study by Seeley E.J. et al. offered a potential answer to this question by showing that local mast cell activation at the infection site is beneficial to the host, while systemic mast cell activation worsens survival during CLP [47]. However, it is still unknown whether CLP of different severities can lead to local versus systemic mast cell activation. 

## 4. Mast Cells and *Candida albicans* Infection

Fungi are associated with many different types of diseases in humans. However, very few research studies have been conducted on the role of mast cells in antifungal defense, especially when compared with the abundance of research on their roles in bacterial, viral, and parasitic infections [73]. Fungi, just like other pathogens, enter the body via mast cell-rich organs, such as the skin, gut, and airways. Therefore, mast cells likely respond to fungi because of their strategic location at vascularized mucosal surfaces [74] and because they express several receptors and mediators known to be involved in antifungal responses [75,76]. 

*Candida albicans (C. albicans)* is an opportunistic dimorphic fungus that causes mucocutaneous and systemic candidiasis [77]. *C. albicans* usually colonize humans at birth and persist throughout life as a commensal in the skin and the oral, gastrointestinal, and vaginal mucosae [78]. The immune response plays an essential role in the control of opportunistic infections by *C. albicans*, as alterations in anatomic barriers or immunosuppression favors candidiasis disease development [79]. In immunocompetent people, candidiasis is usually a localized infection of the skin or more often of the mucosal membranes, including the oral cavity (thrush), the pharynx or esophagus, the gastrointestinal tract, the urinary bladder, or the genitalia. 

**Figure 3:**
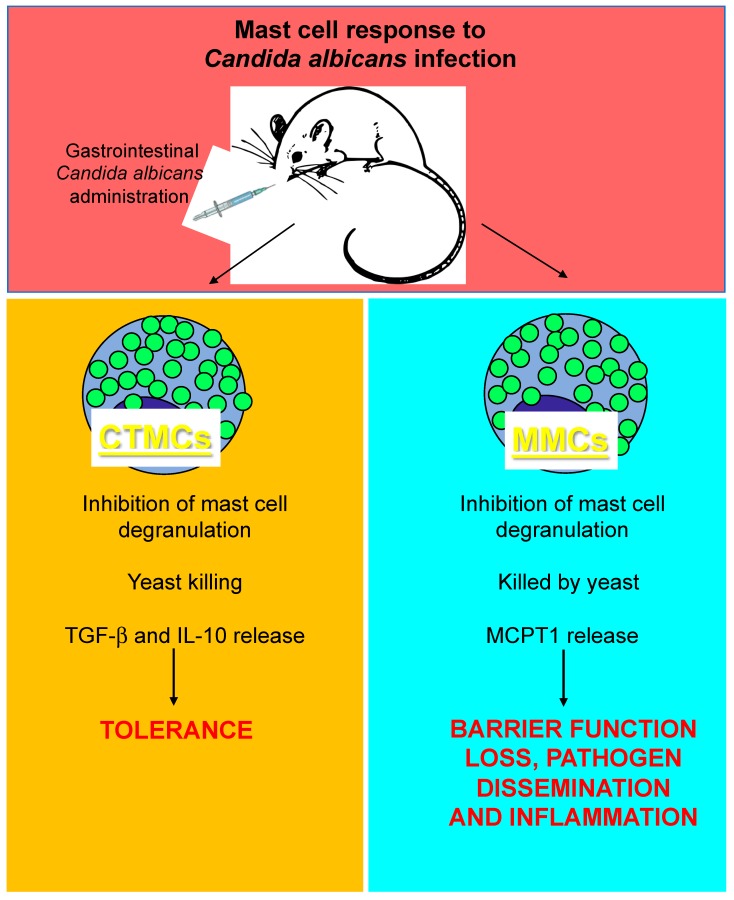
Differential response of mucosal (MMCs) and connective tissue mast cells (CTMCs) to *Candida albicans* infection in the gut. MCPT1, mast cell protease-1; and TGF-β, transforming growth factor beta.

Studies have suggested that mast cells participate in a number of ways in *Candida*/host interactions at mucosal surfaces [80,81]. Murine mast cells phagocytose *C. albicans*, produce nitric oxide by mechanisms involving TLR2 and Dectin-1 [82], and kill the fungus through secreted granular components [83]. In vitro, human mast cells mount a specific series of responses towards *C. albicans*, which initially includes degranulation, neutrophil recruitment, and reduced fungal viability, followed by the release of anti-inflammatory mediators, such as IL-1ra [80]. A detrimental role for mast cells during *C. albicans* has also been proposed. A report by Yamaguchi N. et al. suggests that gastrointestinal *Candida* colonization promotes sensitization against food antigens, at least partly due to mast cell-mediated hyper-permeability of the gastrointestinal mucosa in mice [84]. Overall, this evidence suggests that, as with viral and bacterial infections, mast cells can play beneficial or detrimental roles during *C. albicans* infections. Recent studies seem to support this hypothesis by showing that mast cell interactions with *C. albicans* in the gastrointestinal tract represent a model in which infection outcomes depend on which type of mast cell interacts with the pathogen. Specifically, although both mucosal and stromal mast cells can phagocytose unopsonized yeast and expand in the stomachs of *C. albicans*-infected mice [85,86], they exhibit different candidacidal activity. As previously suggested [83], mucosal mast cells are unable to kill phagocytosed fungi. On the contrary, phagocytosed fungi can kill mucosal mast cells, leading to a massive MCPT1 release, which may contribute to barrier function loss, fungal dissemination, and inflammation in experimental leaky gut models. In contrast, stromal mast cells can kill phagocytosed yeast. Additionally, stromal mast cells produce higher concentrations of transforming growth factor (TGF)-β and IL-10 than mucosal mast cells in response to fungal hyphae, which contributes to increased mucosal immune tolerance [87] (Figure 3). Similar pro-inflammatory versus tolerogenic roles for mucosal and stromal mast cell subsets, respectively, were observed in vulvovaginal candidiasis [88], indicating that the dynamics of mast cell-*C. albicans* interactions described for infections in the gastrointestinal tract can also be observed in other tissues. 

## 5. Concluding Remarks

Recent findings in the field of mast cells and infection seem to support the hypothesis that mast cells can either promote host resistance to infection or contribute to a dysregulated host response that can increase morbidity and mortality. The factors that determine whether mast cells will respond to infection in one way or the other are unclear. However, in the three models examined in this review there seems to be a pattern in the effects of local versus systemic mast cell response on infection outcome: while local mast cell activation at the infection site contributes to pathogen clearance, systemic mast cell activation promotes barrier function loss, inflammation, and pathogen dissemination with a consequential increase in host morbidity and mortality. Another factor that must be considered, although it is less investigated, is the site-specific heterogeneity of mast cells in tissues compromised by an infection and/or host response. The possible influence of this additional factor on disease outcome is supported by the fact that mast cells from different locations and/or phenotypes exhibit a differential profile of mediators that can impact the host during infection. Novel genetic and/or pharmacological approaches to target different mast cell phenotypes in tissues affected by the infection and/or host response will be required to investigate this further.

Genomic and proteomic approaches used by us and others have the potential to provide a more comprehensive understanding of mast cell function in health and disease. However, most of these studies have been focused on the characterization of mast cell genomic or proteomic profiles in the context of IgE-dependent responses relevant to allergy. Considering the studies reviewed here, we think it is important to point out that the mast cell local and systemic responses can exhibit different features depending on the nature of the stimulus. Therefore, comprehensive genomic or proteomic analyses of mast cells in response to stimuli that are relevant to different pathogen infections need to be performed. Importantly, the use of in vivo models of disease has clearly shown that understanding interactions between mast cells and other immune and non-immune cells is also critical and indispensable to our ability to elucidate pathophysiological mechanisms that lead to certain mast cell functions and infection outcomes.
